# Relationship between oral environment and frailty among older adults dwelling in a rural Japanese community: a cross-sectional observational study

**DOI:** 10.1186/s12903-019-0714-8

**Published:** 2019-01-22

**Authors:** Yoko Hasegawa, Ayumi Sakuramoto, Hideyuki Sugita, Kana Hasegawa, Nobuhide Horii, Takashi Sawada, Ken Shinmura, Hiromitsu Kishimoto

**Affiliations:** 1 Department of Dentistry and Oral Surgery, Hyogo College of Medicine, 1-1 Mukogawa-cho, Nishinomiya, Hyogo 663-8501 Japan; 2 Division of Comprehensive Prosthodontics, Graduate School of Medical and Dental Sciences, Niigata University, 5274, Gakkocho-dori 2-bancho, Chuo-ku, Niigata, 951-8514 Japan; 3 Division of General Medicine, Department of Internal Medicine, Hyogo College of Medicine, 1-1 Mukogawa-cho, Nishinomiya, Hyogo 663-8501 Japan; 4 Hyogo Dental Association, 5-7-18 Yamamoto-dori, Chuo-ku, Kobe, Hyogo 650-0003 Japan

**Keywords:** Frailty, Oral health, Salivary bacteria, Elderly

## Abstract

**Background:**

Oral functions are known to decline with aging. However, there is limited evidence that supports the relationship between oral health and frailty. This study aimed to clarify the relationship between oral hygiene conditions, measured by remaining teeth and mucosa, and frailty among elderly people dwelling in a Japanese rural community.

**Methods:**

We surveyed self-reliant elderly individuals aged ≥65 years who were dwelling in the Sasayama-Tamba area of Hyogo, Japan. Frailty was evaluated according to the total score of the Kihon Checklist (KCL). Based on the KCL score, elderly participants were divided into three groups: robust, pre-frail, and frail. The items measured to evaluate oral environment included the number of remaining teeth, denture usage condition, oral hygiene status, dry mouth condition, and salivary bacterial count. For statistical analysis, Fisher’s exact test, one-way analysis of variance, and multiple comparison technique were used.

**Results:**

Of 308 elderly participants, 203 (65.9%), 85 (27.6%), and 20 (6.5%) belonged to the robust, pre-frail, and frail groups, respectively. The proportion of participants who were judged to have poor hygiene was significantly higher in the frail group than in the other two groups. The bacterial count was significantly smaller in the frail group than in the robust group, and the frail group had fewer number of remaining teeth than the other two groups, suggesting that the number of remaining teeth may be associated with bacterial count.

**Conclusion:**

In elderly adults, physical frailty may affect the oral hygiene status and condition of the remaining teeth.

## Background

In aged societies such as Japan, there is an urgent need for protocols that outline the appropriate management of age-related diseases and long-term care associated with disability. The provision of appropriate interventions and care for elderly people at the onset of frailty has been shown to have an important role in preventing the progression of disability [1, 2]. Similarly, preventing the progression of frailty requires interrupting and slowing the factors that contribute towards it, as these contributing factors are then worsened by frailty itself. For example, a lower nutritional status has been reported to contribute towards the progression of frailty [3, 4], and a decline in oral functions worsens frailty [5, 6]. Considering that nutrition contributes to oral environment, maintenance of oral functions may affect frailty. Oral dysfunction contributes to poor eating habits in elderly people, as functions of mastication and swallowing have been shown to decline [7], and a reduced desire or ability to eat leads to lower nutritional status. Oral dysfunction is caused by various factors, including tooth or dental occlusion loss, periodontal disease and dental caries, inappropriate removable or complete dentures, and dry mouth and lingual movement disorder [8, 9]. Some of these factors are closely related to oral hygiene, and thus maintaining good oral hygiene status is considered important for maintaining oral function to prevent the progression of frailty [5, 7]. However, there is limited evidence for the relationship between the maintenance of oral functions and frailty.

Therefore, this study aimed to identify a relationship between oral hygiene conditions, as measured by status of remaining teeth and mucosa, and frailty among elderly people dwelling in a Japanese rural community and assess the association between individuals with potential frailty and their oral health status.

## Methods

This cross-sectional study was approved by the institutional review board of Hyogo College of Medicine (approval no. Rinhi 0342) and is part of the Frail Elderly in the Sasayama-Tamba Area (FESTA) study. The purpose of the FESTA study was to clarify associations between lifestyle habits and frailty in elderly people. The Hyogo College of Medicine Sasayama Medical Center is a branch hospital of the Hyogo College of Medicine, located in the Tanba-Sasayama area of Hyogo, Japan. Therefore, this study was conducted in the Sasayama area.

### Participants

Community-dwelling individuals aged ≥65 years were recruited between June 2016 and December 2016 from the Sasayama-Tamba area, a rural area in Hyogo, Japan. The area is a mountainous region where many people work as farmers. It has a population of 41,490, and an aging rate of 32.6%, compared to the 2015 aging rate in urban Japan of 26.7%.

We recruited participants for this study by distributing newspaper inserts and poster advertisements at Hyogo College of Medicine Sasayama Medical Center. Individuals participated in this research voluntarily, and all participants provided written informed consent. All participants in this study were independent elderly individuals who required less than level 1 care based on the long-term care insurance system in Japan [10]. Individuals who displayed decreased cognitive function (Mini-Mental State Examination [MMSE] score < 20) were excluded [11]. The data used in this study were anonymized. All data were masked for analysis. The authors did not have access to the participants’ personal information.

### Diagnosis of frailty

A self-administered questionnaire survey was conducted using the Kihon Checklist (KCL) [12], which consists of 25 questions for screening participants who need preventive health care in Japan. The KCL was not validated in the FESTA study. The KCL covers the following domains: instrumental activities of daily living (ADL), social ADL, exercise, falling, nutrition, oral function, cognitive function, and depression. The participants were asked to provide either a “negative” (score: 1) or a “positive” (score: 0) answer, for a total score of 25. Frailty was determined using the methods introduced by Satake et al.: frail, 8–25 points; pre-frail, 4–7 points; and robust, 0–3 points [12] .

Medical history data were collected on the following 18 items, using closed-ended questions: hypertension, diabetes, hyperlipemia, hyperlipidemia, liver disease, kidney disease, heart disease, asthma, tuberculosis, pneumonia, gastric ulcer, osteoporosis, rheumatism, thyroid, collagen disease, blood disease, stroke, cancer, and other. All participants provided written informed consent prior to administration of the questionnaire. Participants were informed of their right to withdraw from the study at any point without negative consequences or a loss of privileges.

### Evaluation of oral functions

Participants who used a removable denture were instructed to insert the denture at the time of the assessment. The oral function of participants was assessed from 10:00 to 14:00. All oral examinations were performed in a dedicated room in the survey venue; a partition was placed between participants to protect their privacy during the intraoral examination. Any equipment used in this study and that came into contact with patients was disposable and changed for each patient.

Participants’ number of remaining teeth (min, max: 0, 32) and the denture usage condition were assessed while patients were in a reclining chair (Reclining Tilt Chair®, Boutiques, Inc., Tokyo, Japan); LED headlights (Asahi Electric Co., Ltd., Osaka, Japan) were used by the examiner. We used the Clinical Oral Assessment Chart (COACH; Table 1) to evaluate oral hygiene status [13]. COACH is similar to the Revised Oral Assessment Guide (ROAG) by Andersson [14].

**Table 1:** Clinical Oral Assessment Chart.Recreated with reference to the original COACH chart [8]

Clinical Oral Assessment Chart				
Item		**○:No problem**	**△:Cautious**	**×:Problematic**
		Participants continue current care	Caregivers consider asking a specialist for assessment when no improvement is seen	Participants need treatment or intervention by a specialist
Mouth opening		Participants easily open mouth for care	• Participants refuse to open mouth • Caregivers can open mouth manually with 2 fingerbreadths	Caregivers open mouth with < 1 fingerbreadth because of tooth clenching and contracture of temporomandibular joint
Bad breath		None	Caregivers sense bad breath when approaching the oral cavity	Caregivers sense a smell of bad breath in a room
Drooling		None	Decline in swallowing reflex is suspected but no drooling	Drooling (because of decline in swallowing reflex)
Dryness of mouth and saliva		▪ No friction in mucosa on palpation with gloved fingers ▪ Mucosa has saliva	▪ Slightly increased friction, no tendency for the gloved fingers to adhere to the mucosa ▪ Mucosa has little saliva and is sticky	▪ Significantly increased friction, gloved fingers adhering to the mucosa ▪ Mucosa has little saliva and is dry
Teeth and dentures		▪ Clean and no plaque and debris ▪ No mobile teeth	▪ Small amount of plaque and debris ▪ Several mobile teeth but no hindrance to care	▪ Large amount of plaque and debris ▪ Some wobbly teeth
	Oral mucosa	▪ Pink and moist ▪ No dirtiness	Dry and color change such as reddening	• Spontaneous bleeding, ulcer, and candida infection are observed • Airway secretion, desquamated epithelium, and clotting blood are apparent and tightly attached to the mucosa
	Tongue	▪ Moderate filiform papillae present	Extension and loss of filiform papillae (coated tongue and bald tongue, respectively)	
	Lips	▪ Smooth (no cracking)	Cracked and angular cheilitis	
	Gingiva	▪ Tightened (stippling)	Gingiva is swollen and bleeds while brushing	

We examined dry mouth conditions by measuring twice the oral moisture of the dorsum of the tongue and the buccal mucosa using an oral moisture meter (Mucas®, LIFE Co., Ltd., Saitama, Japan) [15] . Scores of ≤27.9 were defined as dry mouth. Moreover, we measured the bacterial count using the dielectrophoretic impedance measurement method (Panasonic Healthcare Co., Tokyo, Japan) [16, 17] [15, 16] and rated the count from 1 to 7 [18]. These indices are Level 1 (bacteria count < 10^5^), Level 2 (bacteria count ≥10^5^, < 10^6^), Level 3 (bacteria count ≥10^6^, < 3.16 × 10^6^), Level 4 (bacteria count ≥3.16 × 10^6^, < 10^7^), Level 5 (bacteria count ≥10^7^, < 3.16- × 10^7^), Level 6 (bacteria count ≥3.16 × 10^7^, < 10^8^), and Level 7 (bacteria count ≥10^8^).

### Data collection and analysis

All statistical analyses were performed using theχ^2^ test, Fisher’s exact test, Mann–Whitney U test, one-way ANOVA, and Bonferroni’s modification. A *p*-value of < 0.05 was considered significant. Statistical analyses were performed with IBM SPSS 22.0 J (IBM SPSS Statistics, Version 22.0.0 for Windows; SPSS, Armonk, NY).

## Results

A total of 308 elderly adults participated in this study; of these, 107 were male and 201 were female, with a mean age of 72.7 ± 7.1 years. Frail and pre-frail participants accounted for 7 and 28%, respectively (Table 2). The number of remaining teeth was significantly higher in the robust group than in the frail group. The salivary bacterial count of the frail group, which had a lower number of teeth, was significantly lower than those of the other two groups, suggesting that number of teeth is associated with bacterial count. There was no significant relationship between frailty and medical history, and medical history had no significant effect on oral condition, with the exception of hypertension: the number of teeth was significantly lower in participants with hypertension (*P* = 0.045, Mann–Whitney U test).

**Table 2:** Summary of participants and oral conditions.Data is presented as mean ± standard deviation. Multiple comparisons between the robust, pre-frail, and frail groups were performed using one-way analysis of variance and Bonferroni’s modification.BMI, body mass index; NS, not significant; a, significant difference between the robust and frail groups; b, significant difference between the pre-frail and frail groups

		Total (308)	Robust group (203)	Pre-frail group (85)	Frail group (20)	p-value
Age (years)		72.7 ± 0.4	72.1 ± 0.5	73.7 ± 0.6	74.4 ± 1.4	NS
BMI (kg/m^2^)		22.3 ± 0.2	22.5 ± 0.2	21.9 ± 0.3	21.6 ± 0.5	NS
Teeth number		20.3 ± 0.5	21.1 ± 0.6	19.3 ± 1.0	16.4 ± 2.3	a
Salivary bacterial count (min, max: 1, 7)		5.1 ± 0.1	5.1 ± 0.1	5.2 ± 0.1	4.6 ± 0.2	a, b
Oral moisture	Tongue	27.6 ± 0.3	27.4 ± 0.3	27.9 ± 0.5	27.8 ± 0.9	NS
	Bucca	29.6 ± 0.5	29.6 ± 0.8	29.5 ± 0.5	30.2 ± 0.8	NS

The proportion of participants who responded as having no problem with “teeth and dentures” was significantly higher in the robust group than in the other two groups (Table 3). A few participants were judged to be cautious (△ in Table 1) or problematic (× in Table 1) with other items, while some were judged to be problematic with only “teeth and dentures” and “dryness and saliva.” That is, several subjects in this study experienced a dry mouth or teeth or their dentures unclean. To evaluate the influence of medical history on oral hygiene items in Table 3, the participants with cautious (△)/problematic (×) condition in “teeth and denture” were statistically significantly recognized with hypertension (*P* = 0.009, χ^2^ test,).

**Table 3:** Association of oral hygiene status with frailty.Relative value to the corresponding clinical oral assessment chart group (number),Chi-square test or Fisher’s exact test.○, no problem; △, cautious; ×, problematic; NS, not significant

	Robust group, % (203)			Pre-frail group, % (85)			Frail group, % (20)			*p*-value
	○	△	×	○	△	×	○	△	×	
Mouth opening	65.9 (203)	0 (0)	0 (0)	27.6 (85)	0 (0)	0 (0)	6.5 (20)	0 (0)	0 (0)	NS
Bad breath	65.9 (199)	66.7 (4)	0 (0)	27.5 (83)	33.3 (2)	0 (0)	6.6 (20)	0 (0)	0 (0)	NS
Drooling	65.9 (203)	0 (0)	0 (0)	27.6 (85)	0 (0)	0 (0)	6.5 (20)	0 (0)	0 (0)	NS
Dryness and saliva	66.5 (183)	60.7 (17)	60.0 (3)	27.3 (75)	35.7 (10)	0 (0)	6.2 (17)	3.6 (1)	40.0 (2)	NS
Teeth and dentures	69.6 (183)	46.3 (19)	25.0 (1)	25.1 (66)	41.5 (17)	50.0 (2)	5.3 (14)	12.2 (5)	25.0 (1)	0.02
Oral mucosa	66.3 (195)	57.1 (8)	0 (0)	27.6 (81)	28.6 (4)	0 (0)	6.1 (18)	14.3 (2)	0 (0)	NS
Tongue	66.2 (186)	63.0 (17)	0 (0)	27.4 (77)	29.6 (8)	0 (0)	6.4 (18)	7.4 (2)	0 (0)	NS
Lips	65.7 (199)	80.0 (4)	0 (0)	27.7 (84)	20.0 (1)	0 (0)	6.6 (20)	0 (0)	0 (0)	NS
Gingiva	66.3 (183)	62.5 (20)	0 (0)	27.9 (77)	25.0 (8)	0 (0)	5.8 (16)	12.5 (4)	0 (0)	NS

## Discussion

There are currently no standardized items and established criteria for evaluating frailty. The most common method uses the Cardiovascular Health Study (CHS) criteria published by Fried et al. [19]. The present study used the KCL to evaluate frailty. The KCL is useful for evaluating frailty, as it shows a moderately significant correlation with the CHS criteria and is known to be associated with other methods [3, 4] . As the KCL is a confirmed measure of patient frailty, it’s inclusion has added value to the Edmonton Frail Scale [20] .

In this study, the frail group tended to have a lower number of teeth. Tohara et al. [21] have reported that number of teeth is associated with bacterial count, and our findings were similar. The correlation coefficient between teeth number and bacterial count was R = 0.45 (*p* < 0.001), suggesting that teeth number is positively correlated with bacterial count.

Elderly individuals with more remaining teeth are reported to be less frail and have better quality of life than edentulous individuals [6]. Poor oral health is common among elderly people and has been associated with chronic diseases and some components of frailty [6, 22] . Typical oral infectious diseases include dental caries and periodontal disease, wherein dental plaque [23]. The dental plaque forms a bacterial coat on the surface of teeth [24]. With insufficient oral care over time, the plaque thickens, increases the risk of oral diseases. Normally, the human oral microflora is predominately comprises Gram-positive bacteria. However, oropharyngeal colonization with Gram-positive bacteria has been associated with infections such as aspiration pneumonia [25, 26] . Therefore, it is necessary to maintain a healthy oral condition in elderly individuals with remaining teeth. As a typical effect of oral medicine on the oral function, it is well known that medication for cardiovascular diseases including hypertension relate to periodontitis [27, 28]. Dry mouth is a frequent side effect of antihypertensive medication. Salivary flow decreases as a side effect of this medication and expects that teeth, and the state of periodontal condition was also getting deterioration. Our results indicate that the conditions of the teeth and denture affect the adherence of contaminating substances such as oral bacteria. In this study, 44.5% of the participants reported having hypertension. Thus, when an elderly individual has hypertension, health care providers should also pay attention to oral hygiene.

There are some limitations of this study. First, due to its cross-sectional design, a causal relationship between oral hygiene and frailty could not be confirmed. A longitudinal study for this purpose is ongoing. Second, we did not include data on current medication of the participants in the analyses of this study, because the information we received from participants was not reliable (e.g. participants did not know the details of their current medication, they did not bring their list of medications to the intraoral examination, or self-declared medication and their list of medications did not match). As several medications used internally can cause thirst, this should be considered in future studies.

## Conclusions

Oral hygiene status and the condition of the remaining teeth might be affected by the degree of frailty of an individual. The bacterial count of the frail group was significantly smaller than that of the robust group, suggesting that the number of remaining teeth is associated with the salivary bacterial count. Therefore, health care providers should carefully consider management of remaining teeth of older adults.

## Abbreviations

ADL: Activities of daily living
CHS: The Cardiovascular Health Study
COACH: The Clinical Oral Assessment Chart
FESTA: The Frail Elderly in the Sasayama-Tamba Area
KCL: Kihon Checklist
